# Community-based referral transportation system for accessing emergency obstetric services in the Rohingya refugee camp during the COVID-19 pandemic in Bangladesh: facilitators and barriers through beneficiaries’ and providers’ lens using a mixed-method design

**DOI:** 10.1186/s13031-022-00485-7

**Published:** 2022-10-10

**Authors:** Mrittika Barua, Sayantan Chowdhury, Avijit Saha, Chand Mia, Stenly Hely Sajow, Malabika Sarker

**Affiliations:** 1 BRAC James P. Grant School of Public Health, Dhaka, Bangladesh; 2 United Nations Populations Fund, New York, USA; 3 Heidelberg Institute of Global Health, Heidelberg University, Heidelberg, Germany

**Keywords:** Sexual and reproductive health, Rohingya, Bangladesh, Second delay, Referral transportation

## Abstract

**Background:**

Delays in seeking timely maternity care from health care professionals are crucial to address among the Rohingya population where many preventable pregnancy-related deaths occur within the camps when care is not sought. To address the challenges related to the referral of emergency and routine Sexual and Reproductive Health and Rights (SRHR) cases, United Nations Population Fund, through its partners, implemented a community-based referral transportation project called Referral hub. This paper presents the barriers and facilitators to the implementation of this referral transportation system from the perspectives of the beneficiaries and providers.

**Methods:**

The research adopted a sequential explanatory mixed-method design. The quantitative phase consisted of a survey among 100 women while the qualitative phase comprised of in-depth interviews with a total of 12 mothers who used the services and key informant interviews with 21 providers.

**Results:**

The barriers identified for referral hub are discordant understanding of emergency, strict gender norms and practices, distrust in providers, poor roads and mobile phone networks. The facilitators are partnership with the community, within and other organizations.

**Conclusion:**

The study observed that the referral hub has a high potential to increase the utilization of SRHR services. Despite the barriers, the facilitating factors show a scope of improvement for these services.

## Background

With the increasing magnitude of forced displacement resulting from conflict, there is a growing global concern about increasing maternal morbidity and mortality in the displaced populations. It is particularly relevant when women and girls of reproductive age, making up more than half of the refugees worldwide, are most vulnerable to poor pregnancy outcomes such as spontaneous abortion, preterm birth, and preeclampsia [1]. In other words, in addition to the psychological and physical distress from displacement and resettlement, refugee women experience long-term maternal morbidity and mortality. One of the primary reasons for poor pregnancy outcomes is the low utilization of maternal and reproductive health services, which worsens during pandemics [2].

Primarily, the lives of refugees living in humanitarian settings are compromised. There is a lack of resources, political instability, violence, limited access to primary health care needs, water, and sanitation, in addition to poor living conditions. For a pregnant woman, this could also mean poor access to maternal and reproductive services due to language difficulties, lack of awareness, cultural and gender norms, financial constraints, among other barriers [3].

Global evidence hints at high maternal mortality in refugee camps, and they are linked with the delays in care [4]. Delays contributing to maternal deaths usually occur at three stages: the decision to seek care, reaching the facility, and receiving appropriate quality care [5]. These delays are worse during pandemics leading to low hospital visits for antenatal care, delivery, and postnatal care [6]. Moreover, due to pandemics, resources are diverted to basic survival needs and infection control over necessary maternal and neonatal care [2]. These outcomes are harmonious for the COVID-19 pandemic for reasons such as fear of infection, lack of availability of transport due to restrictions on movement to minimize the spread of disease, and reduced opening hours of obstetric facilities [7].

Such is the case for the Rohingya population in Bangladesh, identified officially as Forcibly Displaced Myanmar Nationals (FDMNs). They are one of the largest displaced populations globally and are dubbed the “most persecuted minority on earth” by The United Nations High Commissioner for Refugees (UNHCR) [8]. Around more than 911,566 Rohingyas live in different camps at two remote sub-districts of Cox’s Bazar [9, 10] and receive health care from about 200 health facilities. However, most facilities are not open 24h a day [11], which is problematic given how maternal health is at risk when referral centers cannot offer services for obstetric emergencies 24h a day [12].

Moreover, other barriers hinder the uptake of the vast array of maternal and reproductive services and encourage deliveries and treatments at home. For example, Rohingya women prefer home deliveries for reasons including mixed-gender spaces of facilities, maltreatment experiences at health facilities in Myanmar, and poor road conditions inside the camps [13, 14]. As a result, women stay at home, often for days, with pregnancy-related complications, risking their lives and their child to be born [15].

The COVID-19 pandemic has caused a further decline in the utilization of health facilities in the Rohingya population who have been found to avoid health facilities due to fear of infection and rumors about killing people suspected of COVID-19 besides movement restrictions [16, 17]. Therefore, in such a critical situation, there is a need for transport that can carry emergency obstetric patients to facilities on time, reducing the risk of maternal and neonatal deaths [18].

A systematic review reports several interventions that have been implemented in humanitarian settings to improve access to emergency obstetric and neonatal care [19]. However, such innovations have been implemented at facilities, not community. There is hardly any information on community-based transport interventions. Therefore, the current research presents such an innovation and explores its facilitators and barriers to improve the utilization of emergency obstetric services during the pandemic. The findings can inform future community based transport innovations in a low resource humanitarian settings.

Due to the low uptake of services in mind and anticipation of the first wave of the pandemic, UNFPA implemented the ‘Referral Hub,’ a community-based referral project to provide transport free of cost for emergency obstetric and neonatal care 24/7 through its implementing partner, International Rescue Committee. Given that socio-cultural, economic, and infrastructural barriers already exist within the Rohingya refugee population.

## Method

### Study design

The research adopted a sequential explanatory mixed-method design comprising a quantitative phase (household survey) followed by a qualitative one (in-depth interviews and key informant interviews). An explanatory design was chosen as it helps explain or build upon the quantitative results [20]. A survey was chosen for the quantitative phase to determine the level of knowledge about RH and to quantify the type of experience the female clients had to assess when and how they used the service. Doing so helped us to identify participants with diverse experiences to explore in depth in the qualitative phase. The preferred methods in the qualitative phase were in-depth interviews with mothers and key informant interviews with implementors and providers to understand the experiences related to the service from all the perspectives.

### Study setting

**Fig. 1:**
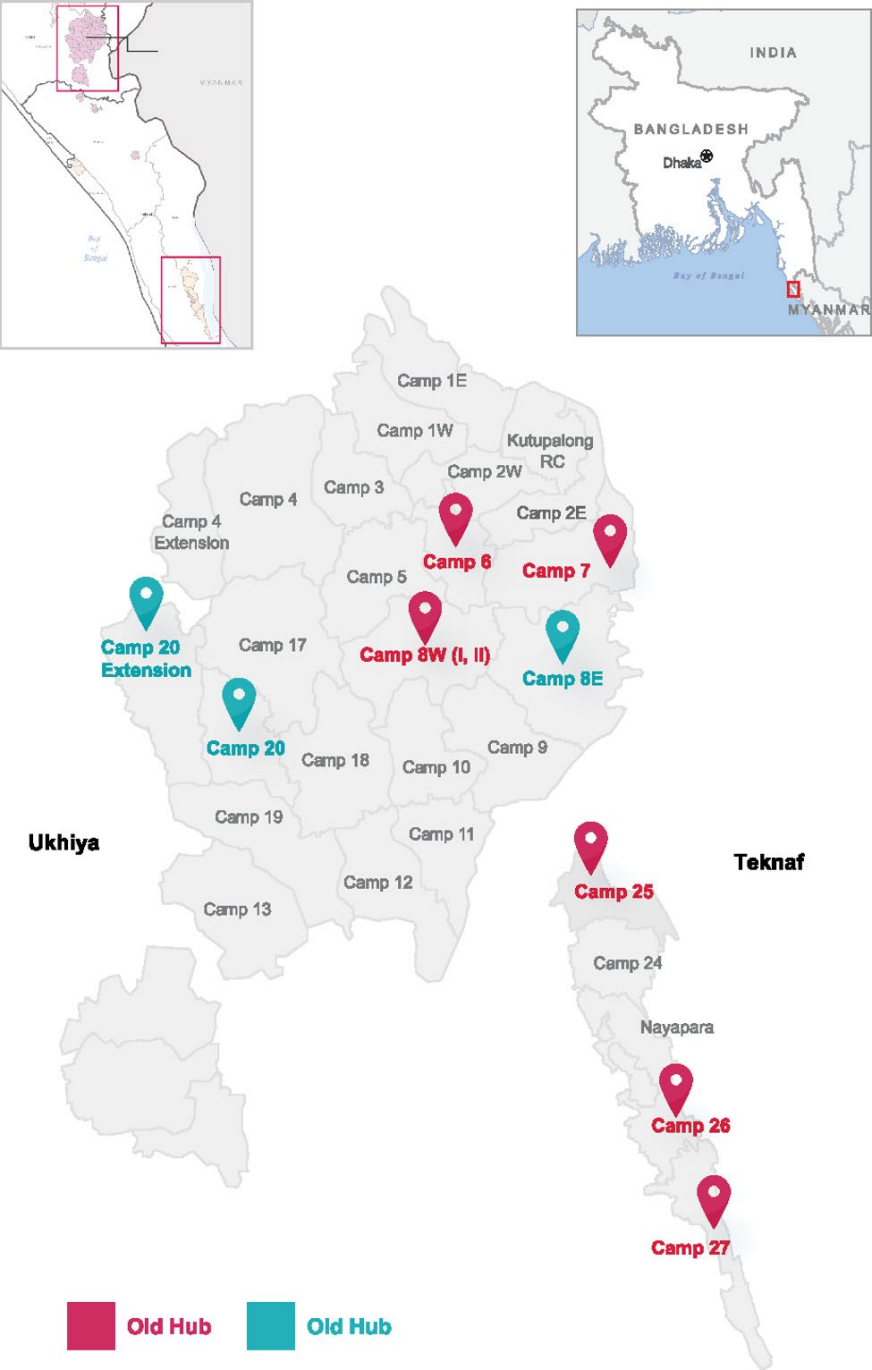
Camps with the Referral Hubs.

The study took place in Teknaf and Ukhia *upazilas* of Cox’s Bazar district in Chittagong. The data collection took place in the camps where the Referral Hub project is being implemented (Fig.1).

### Referral hub transport system

Referral Hubs (RHs) are small structures having cemented floors and roofs made from bamboo and tin. They are built-in hard-to-reach areas with minimal healthcare facilities and a high rate of home deliveries among the Rohingya population. Eight hubs were established in 2019 to address the barriers related to emergency transportation or referrals for emergency obstetric & newborn care. However, in anticipation of the first wave of COVID-19 pandemic UNFPA strengthened the existing referral hubs and expanded into four more hubs in other areas in July 2020. The RHs provide ambulance service for all types of emergencies, particularly prioritizing emergency obstetric and newborn care. Each RH is managed by a Referral hub Team leader (RHTL) who coordinates referrals and supervises the Community Health Volunteers (CHVs), who are men from both host and Rohingya communities. The clients are taken to the facilities in ambulance-like vehicles available 24/7.

**Fig. 2:**
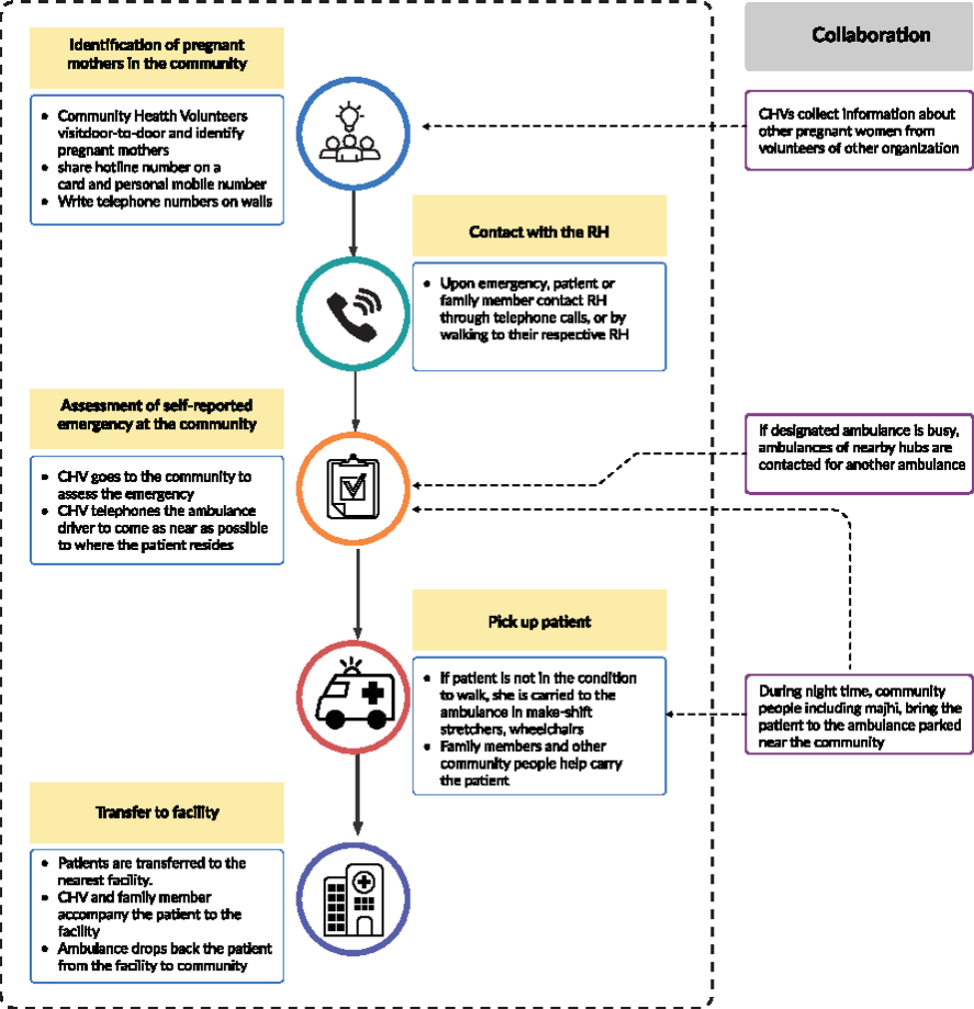
The implementation process of Referral Hub.

A more detailed description of the implementation of the referral hub by the authors is present elsewhere [21]. In short, the RH offers services in the following ways (Figs.2, [19]): First, CHVs visit door-to-door and identify pregnant women in the community. Second, the CHVs share the hotline number of the hub (as well their mobile phone numbers) and inform the women about the free ambulance service and its pick and drop facilities. Once the CHVs come to know of an emergency over the phone or in person, they visit the households to assess the health conditions. Third, after the primary assessment is complete, the CHV calls the ambulance driver. Depending on the physical condition, the patient either walks or is carried to the ambulance. After receiving the service in the nearest health facility, the patient returns to the community by ambulance.

### Quantitative phase

#### Sampling

Since the new hubs were established in July, women who utilized both the old and new hubs in this month were included in the sampling frame. Women who were pregnant during the survey or had delivered their baby six weeks prior were included. A total of 342 women accessed the old and new hubs in this period. Since the number of users varied for each hub in July, we used a proportionate sampling method. This means that the number of women we selected from each hub was proportionate to the overall percentage of women users of that hub. The total number of women selected for the survey via this sampling method was 102. However, the data collectors could reach only 100 as two women in a new referral hub were unavailable.

#### Data Collection

The researchers at BRAC James P. Grant School of Public Health (JPGSPH) of BRAC University could not travel to Cox’s Bazar to collect data due to the lockdown. That is why the survey was conducted by ten field officers (hereafter referred to as data collectors) of Research Training and Management Institute, a partner organization of UNPFA. They received training from the JPGSPH researchers over a virtual platform.

The data collectors identified the mothers in the community with the help of the CHVs and conducted the survey face-to-face at their homes. The survey used a structured questionnaire and focused on knowledge, experience, challenges, and recommendations related to the use of referral hub.

#### Data analysis

Descriptive analysis was conducted to assess the socio-demographic profiles, pregnancy and delivery status of the women, their knowledge of the RH transport, why, how, and when they used it, and their challenges in accessing them. To assess knowledge levels, source of knowledge regarding referral hub transport among all mothers, frequencies of their responses were calculated. To assess the experiences of using the referral hub transport, a comparison was done between those that reported obstetric complications and those that didn’t when they used the transport.

### Qualitative phase

#### Sampling

The survey revealed that some women used the RH transport despite not experiencing any obstetric complications, which was contrary to the criteria to use the service, and hence demanded to be explored more in-depth. Therefore, in-depth interviews were held with women selected purposely from the survey to capture diverse experiences using the transport for ANC, delivery, and PNC services, with or without complications. Given time constraints, 12 women were selected for the interview who were either pregnant or had delivered within six weeks prior to their interviews. This means from each service (ANC, delivery, and PNC), there were two women who reported complications and two who did not. No more women were recruited as data saturation was reached.

The implementing partner IRC provided a list of staff working at the 12 hubs for the key informant interviews (KIIs). From this list, 21 informants were selected to capture staff working at different hierarchies in the implementation. This number of informants comprised an RH manager, four RHTLs, 12 CHVs (one from each hub), and four drivers chosen purposely. Among the CHVs, six were of Rohingya origin and the rest were Bangladeshis. The RHTLs, CHVs, and drivers were from both old and new hubs. No more participants were selected for the KIIs as saturation in data was reached.

#### Data collection

The data collectors who conducted the survey interviewed the women face to face in depth using a semi-structured guide to explore their perceptions and experiences of using the RH service. They also interviewed six CHVs of Rohingya origin. The interviews conducted by the data collectors were face-to-face. JPGSPH researchers interviewed the rest who were of Bangladeshi origin over the telephone. The KII guide had questions to describe the implementation process of the hub and the associated barriers and facilitators of providing the service, and the duties of the informants. Before the interviews, the data collectors received training from JPGSPH researchers over a virtual platform.

#### Data analysis

Transcribers recruited from Chittagong who speaks the local dialect, similar to the language Rohingyas speak, transcribed the audio recordings into Bangla. After the transcripts were read repetitively for familiarization, a content analysis was done via deductive and inductive coding. Deductive coding involved a top-down approach where a pre-set coding scheme was formulated. This means a list of a priori codes (based on literature review and interview guides) was made and applied to the text. After completion of deductive coding, inductive coding was done on new emerging information. This means that as new codes emerged, they were added to the list, and existing codes were modified, when necessary, finally making a codebook with distinct codes with meanings. Once coding was done, the codes were grouped into larger categories in the final stage, and overarching themes and patterns were identified. The themes evolved around the facilitators and barriers in implementation and accessing the referral hub services. Two broad themes were identified: the decision to seek care and reaching the facility. The findings presented in this paper were arranged around these two themes. Quotations from the qualitative interviews have been used throughout to support findings, and participants other than drivers, CHVs and mothers have been categorized as supervisors to protect the identity whenever their quotations have been used.

### Data Triangulation

The qualitative data supported the quantitative findings, showing the results’ convergence and validating the data. The quantitative information gathered from the survey on knowledge, experience, and perception about the service was triangulated with the qualitative data collected from both beneficiaries and providers on experiences.

## Results

### General information of mothers who participated in the survey

Twenty-five mothers out of the 100 were pregnant at the time of the survey, and the rest had given birth in the past six weeks or more. The mothers had a median age of 23 years (Range: 16–35 years) and the majority belonged to the 21–25 years age group (42%). Most mothers reported having at least two children currently alive (29%) (mean: 2.43 children/mother). Most of them lived with their husband (96%) and children (81%) at the survey time. Out of 100 mothers, two reported not knowing “referral hub.” Possibly, they were not familiar with this name even though they have used the service. Therefore, findings from 98 mothers familiar with ‘referral hub’ are presented in the following sections. Seven out of 98 mothers (7.14%) reported not using the vehicle but being referred to facilities by RH staff and included in the analysis. The following sections present qualitative findings on facilitators and barriers under each of the two themes (‘The decision to seek care’ and ‘Reaching the facility’) supported by results from the survey.

### The decision to seek care

#### Facilitators

##### Correct knowledge about RH services

The decision to utilize referral hub services heavily depends on whether clients are aware of the services. The mothers revealed correct knowledge in this respect. The most common response was that the RH provides emergency transport (91%) followed by emergency care (70%). Around 35% and 44% of the women also mentioned that an RH refers to other facilities and provides counselling. Regarding the sources of knowledge, CHV was cited most frequently (94%), followed by neighbours (21%). Community leaders called *majhi* were also mentioned as a source by 2% besides friends and relatives (4%). Therefore, the community is well informed of the hub services, and bringing the community leaders on board helps to disseminate the messages. This quantitative finding is supported by the qualitative finding below.

##### Gatekeepers turned matchmakers: the engagement of community leaders

Having *majhi*, considered a ‘local guardian of the block,’ as a source of information about RH works in favour of RH, as the survey revealed. The qualitative interviews backed this finding too. For example, one informant said,

*Due to our communication with Majhi, we get information about pregnant mothers in this block. Emergency patients usually inform Majhi about their choice of facility. We circulate our hotline number to Majhi so they can notify us immediately. The patient can receive the service quickly. – (Supervisor, 27, IDI 2)*

A good relationship with the *majhis* also enables the emergency transfer of patients at night when no outsider is allowed inside the camp. The *majhis* also help to convince families to go for facility delivery, especially the pregnant women who feel shy to talk to CHVs.

*I got an emergency call at 2 AM., but how can I get into the camp at that time? I cannot go there without Majhi’s confirmation. So, I called him and asked to stay there after confirming the emergency. – (CHV, 28, IDI 6)*

*All family members agree to institutional delivery except the husband. We communicate with majhi, imam, and local elite people. We approach them. Majhi convinces the patient and the guardian of the patient. Then, we refer the patient. – (Supervisor, 28, IDI 3)*

##### Symbiotic relationship with other organizations

Besides majhi, the CHVs work collaboratively with other organizations’ community health workers (CHWs) to find new pregnant women. These CHWs go door to door and collect and share information about pregnant women with the CHVs. Such a liaison also helps the RH staff to reach emergency patients as the CHWs inform them when they find any emergency patient in the community.

*In each block, volunteers of every organization work. We ask them whether they know any delivery patient [and] emergency patient. We promote our services by communicating with [other] CHVs, site management [team], health focal or partner organizations. – (*Supervisor, 29, IDI 13)

*In our camps, we collaborate with community health volunteers of other organizations who work on maternal health, ANC, PNC. Call our hotline number for referral services if they find any emergency patients. – (Supervisor, 28, IDI 3)*

A collaborative relationship between the RHs and the health facilities helps mitigate the shortage of ambulances. Most of the service providers mentioned that they manage ambulances from the neighbouring RH or facilities when they face challenges due to the overload of emergency patients.

*If we find these ambulances unavailable, we contact hospitals. We have a good relationship with them. They also support us by providing their ambulance in emergency cases. –* (Supervisor, 35, IDI 11)

The collaborative relationship between the organizations seems to be favourable as all the women who used the vehicles were satisfied with the service and recommended it for several reasons. The survey result suggests, ‘timely transportation during requirement’ (82%), ‘24/7 availability of transportation (76%), ‘good behavior of the staff’ (59%), and’ staff always available’ (30%) were the main reasons why the mother liked the RH services. Nineteen mothers (21%) also mentioned free service as one reason.

#### Barriers

##### Discordant understanding of emergency

The understanding of *when* to seek hub transport was not homogenous between the beneficiaries and the providers, which was evident in the quantitative and the qualitative phases. Ninety-one women out of 98 (93%) mentioned that they used the transport service—the remaining seven (7%) reported being referred to facilities by RH staff. In addition, all women who reported complications used RH transport. However, 55 (89%) women who reported no complications also used the vehicle, showing that the understanding regarding when (not) to seek transportation was not similar among all women. The narratives revealed that most mothers called for the RH ambulance only when they realized the ‘complication’ required medical treatment. One mother said,

*We told them, “The mother just delivered her child at home. Now she is not feeling well. There is a lot of bleeding. She needs to be taken to the hospital. We do not have any travel fare. Where will we poor people go? If you can provide the service from the number, you gave us, please come”. -* (Mother, *27, delivered, complication after delivery*)

The fact that the mothers prefer to wait until a critical situation arises was also confirmed by some providers. A driver said,

*They do not contact us immediately the minute they feel labour pain. We take in every patient we get. When we would ask them about the time that they started to feel the pain, they would tell us it happened two to three hours ago. We ask them, “Why did you call us so late? You could call us when the pain started”. I don’t understand why they wait to call us. –* (Driver, 30, IDI 14)

**Fig. 3:**
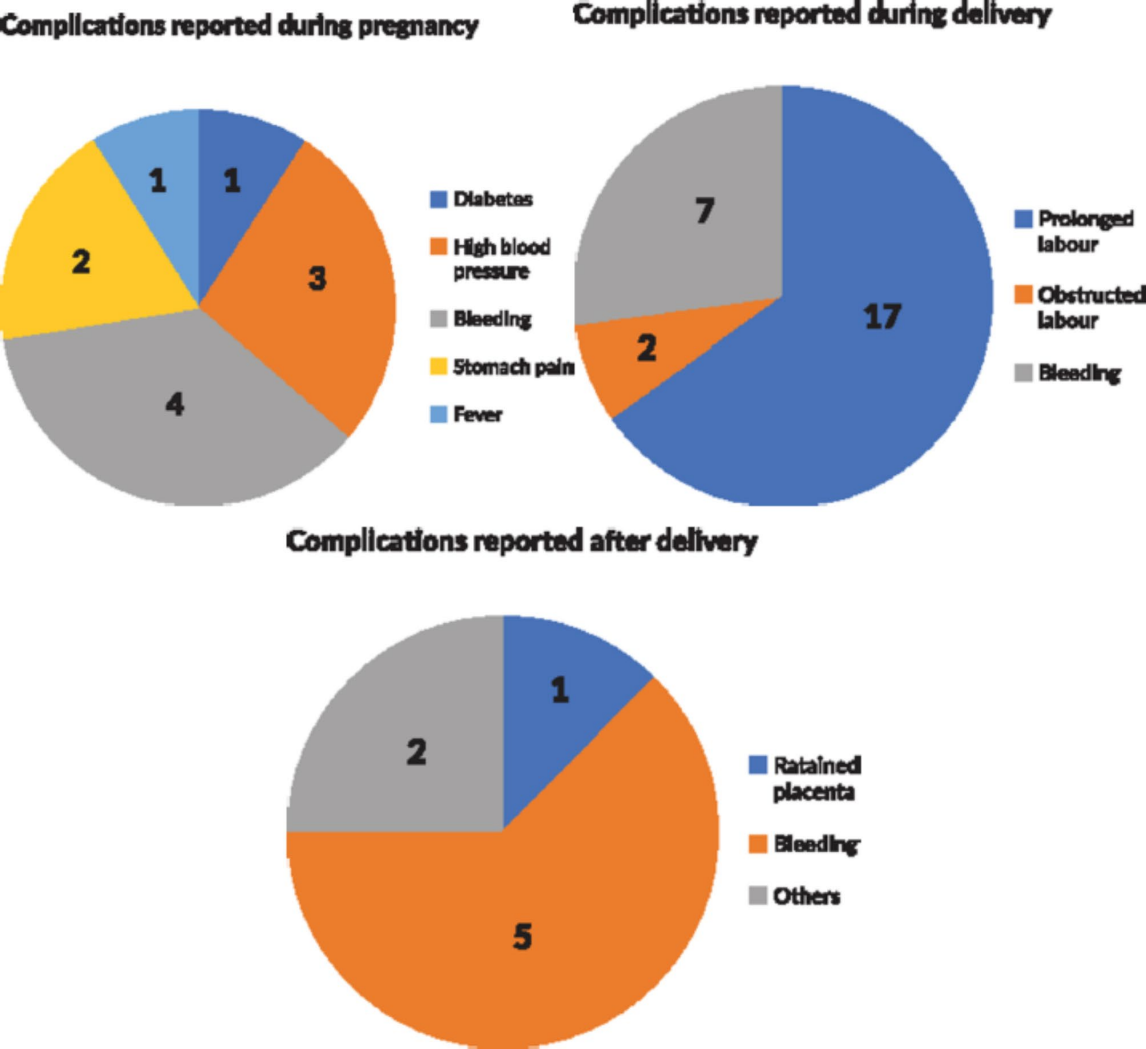
Complications reported by women who were pregnant or had delivered six weeks before the survey.

The above finding can be corroborated by the nature of complications reported by mothers in the survey. As presented in Fig.3, for those ten women pregnant at the time of the study, most mentioned bleeding (n = 4), followed by high blood pressure (n = 3). Women who had already delivered cited prolonged labour the most (n = 17). Seven of them also mentioned bleeding. Eight women reported experiencing complications after delivery. The most common complication after delivery was bleeding. In addition, one woman reported retained placenta as a complication.

Although the providers revealed in the interviews that they expect pregnant women to seek service the moment they start to feel labour pain, the messages they give to pregnant women during household visits tell otherwise.

*We tell the pregnant mothers about the danger signs during pregnancy and that they should immediately go to the hospital when such signs are visible. – (CHV, 27, IDI 8)*

The quotation reveals that the providers expect mothers to seek transport *only* when there is any ‘danger sign’ or any complication and that labour pain is typically not an emergency.

##### Strict gender norms and practices

According to the providers, the social and religious norms to practice veiling and pressure from family and community influence women to give birth at home with the help of traditional birth attendants [TBA], which further worsens complications and makes hospital admission difficult, as revealed in the qualitative interviews.

*There are also cases where mothers go for home delivery, and the traditional birth attendant worsens the complication by trying to treat herself. It becomes challenging because no health facility wants to receive such patients. They say, “why did you bring this patient now? The birth attendant made it worse. Now it becomes more difficult for us to treat”. Now, who knows if the mothers tell us the truth. They say the situation got worse two to three hours ago. But in reality, after you take them to the hospital, you would see that they have been trying to solve the matter at home for a long time which becomes detrimental for both the child and the mother. –* (Driver, 29, IDI 9)

Strict gender norms also prevent the Rohingya women from interacting with the male community health workers. They even avoid going to facilities fearing mixed-gender spaces, vehicles, or facilities. It hampers the task of CHVs, which is to collect pregnancy-related information and take patients to facilities.

*The female members of the Rohingya community are very conservative. They do not come in front of people. Every one of my volunteers is male. In that case, it is tough to take a patient’s medical history. So, they cannot ask the patient directly. They have to take permission from the patient’s husband. Few patients deny talking. CHVs take the information of LMP and EDD after seeing the prescription. – (Supervisor, 27, IDI 4)*

The efforts to talk to the mothers often become fruitless when family members themselves do not allow women to speak to the CHVs. Such a strict gender practice also proves to be a problem when if women have to give birth on the way to the facility. Male CHVs cannot assist the process as no female health worker is available in this situation. One driver explained,

*If a delivery patient cannot walk, we carry her on the stretcher to the ambulance. Sometimes on the way [to the hospital], delivery takes place. We cannot touch the woman as we are all men at that time. Even we do not have a nurse who can accompany us. – (Driver, 30, IDI 14)*

##### Distrust in health facilities

Three key informants cited distrust in health care facilities as a reason for the Rohingya refugees’ refusal to visit health facilities. According to one, the lack of trust is rooted in past bitter experiences in Myanmar. He said,

*When they were in Myanmar, they were not treated well at the hospitals. They fear the same here. That is why they refuse to go. (CHV, 20, IDI 7)*

According to the other two, this fear is even more during the COVID pandemic as the Rohingyas fear getting killed if suspicioned or diagnosed to have COVID.

*They [Rohingyas] fear being tortured at hospitals, or the doctors will not treat them correctly. They have this belief. This is now worse in the pandemic. We came across many cases where they refused to go to the hospitals. They think they will be maltreated even more because of COVID [if suspected or diagnosed with it] ... […] …They believe that the doctors would not care for them as Rohingyas. – (Supervisor, 27, IDI 4)*

### Reaching the facility

#### Facilitators

##### Getting the job done: intra-organizational strategies

The narratives from the providers revealed a strong working relationship among the staff of the referral hubs that facilitates the utilization of the RH transport services. For example, the CHVs keep in touch with their supervisors and notify them immediately of any challenges. Such a relationship is beneficial when CHVs cannot persuade the mothers to give birth at hospitals or assess emergencies.

*Volunteers do counselling. When CHVs find any patients who never become motivated, they inform us. Then, I visit those patients and talk with the family members.” – (Supervisor, 27, IDI)*

*If volunteers are confused about the patients’ category, whether the patient is an emergency or not emergency, they directly call the team leader. They explain the situation of the patient. Team leaders can assess the patients for having medical knowledge and suggest CHVs accordingly. – (Supervisor, 29, IDI 13)*

There is also good coordination between the hubs, which helps to cope with the shortage of ambulances. For example, when two emergency patients need transport at a time, CHVs manage ambulances from other hubs and provide support immediately.

*When we need to provide referral services for more than one patient, we communicate with the referral hub of 25 or 27 to send their ambulance. –* (Supervisor, 35, IDI 11)

#### Barriers

##### Communication delays: poor telephone network

Poor mobile phone network was a common barrier mentioned across all the interviews. Due to the network problem, beneficiaries sometimes cannot contact RH through hotline numbers in time. The RH staff, too, have trouble communicating over the phone with the hospital authority before bringing the patient to the hospital.

*We see the patient’s condition first and immediately take her to the hospital. We communicate with the hospital before going there. Sometimes we cannot contact the hospital due to a network problem. In that case, when we bring the patient without communication, we find that doctors are not available. Then, we have to go to another hospital again. It is a challenge for us. – (Supervisor, 27, IDI 4)*

##### Shortage of ambulances

A shortage in the number of ambulances also contributes to delays in reaching the facility. Usually, one ambulance supports two hubs. Therefore, when there is a high load of emergency patients, the RHs struggle to provide timely service, which is a matter of distress for patients who need immediate transfer to the facility.

*They came and checked everything and said, “give us some time. Another patient took the ambulance. You need to give some time for this”. This was before noon. By the time the ambulance came back, it was already 2 PM.- (Mother, 20, delivered)*

For the same reason, the RH ambulance cannot drop the patients back home from the facility when another emergency patient needs the ambulance.

*We use one ambulance to cover two referral hubs. We prioritize emergency patients to bring them to the hospital. We cannot support back referral from the hospital to home. Unless we find any request from the health sector or doctors, we try to convince the patients to go home on their own. – (Supervisor, 29, IDI 13)*

##### Poor roads: a distressful journey

Poor roads within the camps hamper quick and safe transfer to the facility. The camps are situated in hilly areas with no paved road inside. Therefore, the ambulance drivers need to drive slow and carefully on the slopes. What is worse, the streets become muddy during the rainy season, making it even more difficult for the CHVs to carry the patient causing further delay.

*It has been raining continuously for the last five days. The roads have become muddy. It is not easy to walk. When we pick a mother, it is dangerous to bring her on a stretcher because someone’s foot can slip [while walking]. This is a big challenge… […] …Sometimes, the ambulance wheels get stuck into the mud. It happens every day.* – (Supervisor, 35, IDI 11)

The poor road conditions added to the existing physical distress of mothers suffering from complications. One of them shared,

*If only the ambulance could come to my house, then my suffering would have been less. - (Mother, 27, pregnant, felt complications during pregnancy)*

##### Lack of security

Lack of security at the camps is a crucial challenge that hampers how quickly the RH staff can take the emergency patient to the facility. When patients need to board the ambulance, the drivers often have to do security checks enforced by their authority. Therefore, there is further delay. One driver shared,

*While boarding the patient inside the ambulance, we make sure we search all those bags if people around the ambulance carry bags. I did not face any incident, but we have heard from others that sometimes these people pretend to be fake patients to traffic things illegally. – (Driver, 38, IDI 1)*

During the night, the RH staff become more conscious about their security due to ongoing conflicts in the camps. There are incidents of gun shooting and killing inside the camps as one driver shared,

*It is scary at night to go inside the camp. There are multiple problems. Often there is shooting, sometimes people are killed, and a dead body is left on the roads. In this situation, two volunteers of RH go to the camp at night along with me... So, a total of three people. But in daytime one volunteer and I (driver). There is no extra staff.* – (Driver, 30, IDI 14)

The feeling of lack of security also extends to the ambulances. The drivers sometimes cannot help the volunteers carry patients as they are afraid of someone vandalizing the ambulance while they are away.

*When we bring a patient, we are scared of children. Anytime they (children) can throw bricks at the ambulance while stationed.*

To summarise, with respect to the decision to seek care, the facilitators identified are correct knowledge about RH services, engagement of community leaders, and collaboration with other organizations while the barriers are discordant understanding of emergency, strict gender norms and practices, and distrust in health facilities. Regarding the second theme ‘reaching the facility’, the facilitator that came up was intra-organizational strategies and barriers were communication delays due to poor telephone network and roads, shortage of ambulances, and lack of security.

## Discussion

The current study identified several facilitators and barriers to implementing the RH corresponding to the three-delay model’s first and second delay [5]. Overall, the key aspect of RH that facilitates its implementation is the partnership at various levels, with the community, other organizations, and within the same organization. Such alliances and connections help reach out to women who need emergency transport services and ensure access to the facility when needed. For example, the *majhis* were identified in the current study as key players in motivating mothers and their families to use the service, especially when there is an obstetric complication. They also helped carry emergency patients to vehicles, especially at night, when camps are not safe for outsiders.

Similarly, collaborating with other organizations’ volunteers working in the same area is beneficial since pregnant women hesitate to talk to CHVs due to the strict gender norms and veiling practices. In addition, an intra-organizational collaboration between the referral hubs guarantees the provision of an ambulance whenever there is a shortage. Such partnerships are crucial since the Rohingyas are reluctant to seek emergency obstetric care or cannot reach the facility on time due to poor roads and transport systems.

Partnerships are especially vital during pandemics when resources for preventive measures are prioritized. Primarily the COVID-19 pandemic has fueled food insecurity and reduced income in the Rohingya population [22], which may further lead to de-prioritization of health care. Moreover, the Rohingyas are reluctant to visit health facilities due to fear of infection. In this context, intervention strategies such as the provision of free transport must be such that they are acceptable and easily accessible to mothers. This is where community engagement and partnerships play a huge role in enhancing the acceptability and accessibility of a community-based referral service.

Effective partnerships between different stakeholders are valuable for the success of community-based interventions, as shown by research globally [23, 24]. For example, providing transport backed up by strong community partnerships has increased acceptability and utilization of maternal and neonatal services at facilities and prevented pregnancy-related complications in Mozambique, Ghana, and Pakistan [23, 25, 26]. Therefore, continuous partnership with community leaders and other organizations is sorely needed to implement RH services successfully. In a way, by involving the community leaders and the community, the referral hub addresses the fourth delay mentioned in the literature, which occurs when there is no collective action in the community to enable the pregnant mother to reach the facility [27].

However, despite the facilitators, a couple of barriers hamper the referral hub’s smooth implementation. One of the most crucial challenges is the strict gender norms and veiling practices of women in the community, leading to a strong choice for home deliveries [28]. Rohingya women are reluctant to give birth at facilities that have mixed-gender spaces, as reported in a review article [13]. Therefore, it is possible that Rohingya women are not allowed to or may feel uncomfortable travelling in the RH vehicle, which is a mixed-gender space. This may be why they try to deliver at home first instead of going to the facility using RH transport, as found in the current research. In addition, Rohingya women do not interact with men in public, making it difficult for male CHVs to collect pregnancy-related information. Therefore, such a barrier may delay timely emergency transfers to facilities, putting the mother and the newborn at risk of complications and death. These findings also suggest that perhaps having a female volunteer instead of a male volunteer to collect pregnancy-related information and accompany the mother to the facility to give birth could encourage more deliveries at facilities.

Besides strict gender norms and practices, the key informants also reported distrust in health care providers as another barrier to utilizing RH service, a finding mentioned elsewhere as well [13]. The lack of trust among Rohingya refugees could be rooted in denial of citizenship and violations of basic human rights, particularly those concerning sexual and reproductive rights for women, along with providers’ denial of care and violence at health facilities in Myanmar [29]. However, this finding was reported by the providers only. Future studies should interview women and their families in-depth to unravel whether distrust in health providers plays a role in the lower uptake of health services as it has elsewhere [30].

Another significant barrier in seeking care is the discordant understanding of emergencies between pregnant mothers (or their families) and the providers. The interviews with the providers revealed that they expect mothers to call when labour pain starts as they want the mothers to give birth at facilities. However, this was contradictory to the messages they convey to pregnant women, which primarily focus on pregnancy’s danger signs that demand an urgent transfer to facilities. Therefore, it is no surprise that most women sought RH service when their complications were severe and beyond their treatment ability. Moreover, due to differential understanding, it is unclear whether women who reported no complication had any or to what degree. Future studies should explore in-depth what mothers understand by emergency and what messages they receive from the health providers to corroborate their practices and choices of delivery. This is important because unclear and inconsistent instructions may delay receiving care and put women at risk of dire maternal and neonatal outcomes. Unless both the clients and the providers have a similar understanding of when mothers should seek service, there will always be a gap in service provision.

Concurrent with previous findings, barriers such as poor mobile networks and dreadful road conditions also hampered quick care [11, 13]. The nature of settlements and their location already compromise a client’s transfer to a facility and would continue to induce delays in seeking care. Although clients are carried on stretchers or wheelchairs to vehicles, dangerous soil erosion and landslides are risky.

Several limitations to the study exist. Firstly, time constraint was a big limitation as implementors needed quick feedback on the program implementation for adaptation. Moreover, due to the time limitation, not all mothers could be surveyed as intended to. A bigger sample size would have been better to measure the outcomes more precisely and to capture more diverse participants to interview in depth in the qualitative phase. Secondly, the training and supervision needed to be done online over a virtual platform as the JPGSPH researchers could not travel to the field site due to the pandemic. An in-person training and supervision would have enabled the team to understand the context better and address field-related challenges. Thirdly, the language barrier may have hindered the mother from sharing their experiences in great detail. Finally, the fact that the data collectors were male may have discouraged the women from talking about their pregnancy in-depth.

Nevertheless, the study observed that the free transport provided by RHs enabled women in the Rohingya community to seek emergency and routine services from SRHR facilities, particularly during the COVID-19 pandemic. Given that the camps are placed precariously on hilly areas with narrow roads and terrains that become even more dangerous when it rains, the transport service is a blessing for a pregnant mother with an emergency or who cannot walk. The current research shows that many women who sought RH care had complications. Therefore, this implies a greater chance of more deaths without such an intervention. Since there is a drastic reduction in the utilization of maternal health services due to the COVID-19 pandemic, and even more so in the Rohingya population, other programs could also implement such an intervention to improve utilization. The mothers could access ambulatory services and reach the health facilities on time. The current study implies the dire need for exploring the barriers and facilitators of innovations to improve the utilization of facilities for emergency obstetric care in a low-resource humanitarian setting. Given the acceptability of the transport service, the study hints that addressing the barriers would improve the services and increase uptake.

The current research has several recommendations. Firstly, female health workers should be deployed in the community to disseminate messages related to pregnancy, and to accompany mothers to the facilities to avoid reluctance of being in mixed-gender spaces in the vehicle. Secondly, midwives should be engaged in community mobilization to motivate family members to admit women to facilities when an emergency arises. Thirdly, incentives could be provided to traditional birth attendants to refer pregnant women to health facilities for delivery or seeking care for complications. Fourthly, mothers or families of mothers who have used the RH service could be used to encourage other women and their families. Lastly, and most importantly, messages disseminated to women should be revisited so that women know they should seek care when they have labour pain, not only when danger signs arise.

## Conclusion

The RH project enabled mothers to seek emergency obstetric care during the COVID-19 pandemic. The current research shows that a community-based transport innovation can improve access to facility obstetric care in low-resource and humanitarian contexts through a strong referral mechanism by collaborating with the community and arranging free pick-and-drop transport services.

## Data Availability

The datasets used and analyzed during the current study are available from the corresponding author on reasonable request.

## Abbreviations

ANC: Antenatal Care
CHV: Community Health Volunteer
FDMN: Forcibly Displaced Myanmar Nationals
PNC: Postnatal Care
RH: Referral Hub
RHTL: Referral Hub Team Leader
UNHCR: United Nations High Commissioner for Refugees
